# A Systems Perspective on Lifestyle Behaviors, Excess Weight, and Healthy Longevity in the European Union

**DOI:** 10.3390/nu18142275

**Published:** 2026-07-11

**Authors:** Anca Antoaneta Vărzaru

**Affiliations:** Department of Economics, Accounting and International Business, Faculty of Economics and Business Administration, University of Craiova, 13 AI Cuza Street, 200585 Craiova, Romania; anca.varzaru@edu.ucv.ro; Tel.: +40-7-7392-1189

**Keywords:** healthy dietary behavior, fruit and vegetable consumption, health-enhancing physical activity, lifestyle risk factors, sugar-sweetened soft drinks, overweight and obesity, population health outcomes, European Union

## Abstract

**Background/Objectives**: Healthy eating and regular physical activity support chronic disease prevention and healthy aging, while smoking, sugar-sweetened soft drink consumption, and excess weight remain important public health concerns in Europe. Nutritional status and lifestyle behaviors are also relevant to the progression and management of obesity-related metabolic and degenerative diseases, which justify a broader, systems-oriented analysis of lifestyle–health profiles. This study examines the associations between lifestyle behaviors, excess weight, and population health outcomes across European Union Member States and explores whether Member States exhibit distinct lifestyle–health profiles. **Methods**: The analysis uses cross-sectional data from the European Health Interview Survey for the 27 European Union Member States, along with related Eurostat indicators. Healthy dietary behavior is measured through daily fruit and vegetable consumption. The study also includes health-enhancing physical activity, daily smoking, sugar-sweetened soft drink consumption, overweight and obesity prevalence, life expectancy, and healthy life years. Pearson and Spearman correlations, reduced linear regression models, hierarchical clustering, K-means clustering, and cluster-based comparisons are used. **Results**: Healthy dietary behavior was positively associated with life expectancy (r = 0.459, *p* = 0.016), as was health-enhancing physical activity (r = 0.435, *p* = 0.023), while overweight prevalence was negatively associated with life expectancy (r = −0.505, *p* = 0.007). The reduced regression model, including healthy dietary behavior, physical activity, and overweight prevalence, explained 47.4% of the variance in life expectancy. Cluster analysis identified three distinct lifestyle–health profiles: a healthy-diet, low-excess-weight and high-longevity profile; a lower-diet-quality, higher-smoking and lower-health-outcome profile; and a mixed profile characterized by low smoking, higher excess weight, and relatively favorable health outcomes. **Conclusions**: The study supports a systems-oriented view of lifestyle and population health in Europe. Its findings may inform more targeted nutrition, obesity prevention, and public health policies across EU countries.

## 1. Introduction

Non-communicable diseases remain a key issue in current public health, especially in aging European societies. Chronic ailments have long-term effects on the healthcare system, employment productivity, social inclusion, and quality of life. The World Health Organisation has frequently emphasized that prevention involves ongoing attention to modifiable behavioral factors, including diet, physical activity, tobacco use, alcohol intake, and body weight [1,2,3]. In the meantime, health is not solely a matter of individual decision. It also reflects social, economic, cultural, and institutional constraints that affect access to nutritious food, opportunities for physical activity, preventive care, health literacy, and everyday living environments. This broader perspective aligns with research on the socioeconomic determinants of health and enduring health inequalities in Europe, which shows that behaviors are often shaped by deeper disparities in income, education, social standing, and policy environments [4,5,6].

From this perspective, nutritional status and lifestyle behaviors are relevant not only for disease prevention, but also for the progression and management of obesity and metabolic disorders [1,2]. Poor diet quality, excess body weight, physical inactivity, smoking, and regular consumption of sugar-sweetened beverages may contribute to metabolic dysregulation, inflammatory burden, insulin resistance, cardiovascular risk, and functional decline [1,7,8,9]. Conversely, improved dietary patterns and regular physical activity can support weight management, metabolic control, and healthier aging [3]. These relationships justify examining lifestyle indicators, excess weight, and population health outcomes as interconnected components of a broader public health system.

Within this approach, diet behavior is a key determinant of population health. Diets high in fruits and vegetables have long been linked to reduced risks of cardiovascular disease, certain malignancies, and all-cause mortality [7,8,9]. International cancer prevention recommendations and global burden assessments also put diet at the core of chronic disease prevention, revealing that inadequate dietary patterns contribute substantially to preventable morbidity and mortality [10,11]. From a systems perspective, dietary behavior cannot be reduced to a matter of human taste, as food consumption depends on availability, cost, cultural customs, dining practices, and public policy. Hence, previous research has argued that nutrition policies need to target both individual behavior and broader food systems, especially in circumstances where poor dietary habits intersect with socioeconomic inequality and commercial determinants of health [12,13].

Physical exercise is another important element of health promotion, but should not be easily subsumed under food behavior as a single empirical construct. Since the seminal study by Morris et al. [14], evidence has repeatedly shown that physically active lifestyles are associated with reduced mortality and increased life expectancy [15,16]. Physical inactivity is a key contributor to the burden of major non-communicable illnesses, according to Lee et al. [17]. Higher levels of moderate physical activity may mitigate the detrimental effects of sedentary behavior on mortality [18]. However, physical activity may be subject to distinct population-level social and cultural trends than fruit and vegetable eating. Hence, this study considers healthy dietary behavior and health-enhancing physical exercise as two distinct yet interconnected lifestyle characteristics.

Overweight and obesity are closely linked to behavioral patterns and population health outcomes. Excess weight results from complex interactions among diet, physical activity, socioeconomic factors, food systems, metropolitan surroundings, and broader lifestyle routines. The global prevalence of overweight and obesity has been well documented, and obesity is increasingly viewed as a complicated public health problem rather than only an individual energy imbalance [19,20]. The Global Burden of Disease framework also indicates that high body mass index is a major metabolic risk factor for death and disability-adjusted life years worldwide [21]. In Europe, smoking, obesity, and alcohol intake have jointly shaped the changes in life expectancy, illustrating the combined and historically inconsistent impact of lifestyle-related risks across countries [22].

Lifestyle risk factors such as daily smoking and intake of sugar-sweetened soft drinks also warrant investigation not only as individual predictors of health outcomes, but also as markers of larger lifestyle–health profiles. Their effects might not always be evident in straightforward linear relationships in a cross-sectional country-level analysis, particularly when only 27 EU Member States are considered. However, these behaviors may help identify less desirable behavioral configurations, especially when considered alongside dietary habits, physical activity, excess weight, life expectancy, and healthy life years. That is why a systems-oriented approach is useful: it allows the study to go beyond isolated associations and to explore how different behavioral and health variables cluster across countries.

The present study aims to explore the associations between healthy dietary behavior, health-enhancing physical activity, daily smoking, sugar-sweetened soft drink consumption, excess weight, and health outcomes in the European Union population across the 27 Member States, using data from the 2019 European Health Interview Survey and other Eurostat health indicators. The study used an exploratory comparative strategy, suitable for country-level cross-sectional data and not assuming a strong causal structure. It starts with the examination of bivariate relationships among major lifestyle and health variables. Tests reduced regression models for life expectancy and healthy life years, and end with the identification of different lifestyle–health typologies of EU countries through cluster analysis. It thereby adds to the research on nutrition, lifestyle risks, obesity, and population health by providing a systems-based map of behavioral and health heterogeneity within the European Union.

## 2. Literature Review and Hypotheses Development

### 2.1. Healthy Dietary Behavior and Population Health Outcomes

Healthy dietary behavior is an important component of public health because everyday food choices are connected with long-term morbidity, mortality, and healthy aging [1,2,7,8,9]. In this study, healthy dietary behavior is defined through daily fruit and vegetable consumption, two indicators commonly used in population-level nutrition research and public health monitoring [7,8,9,11]. Although diet quality also includes whole grains, legumes, nuts, fish, sodium, added sugars, processed foods, and total energy intake [11,12,23,24], fruit and vegetable consumption remains one of the most comparable markers of healthier dietary patterns in cross-country analyses. Nevertheless, nutritional behavior should be understood more broadly than just fruit and vegetable intake. It also includes overall dietary patterns, meal timing, breakfast consumption, beverage choices, sleep-related eating behaviors, and interactions between sleep and nutrition. These dimensions may shape energy balance, appetite regulation, metabolic health, obesity risk, and long-term population health outcomes. In the present study, daily fruit and vegetable consumption is therefore used as a harmonized country-level proxy for healthy dietary behavior, rather than as a comprehensive measure of nutritional behavior or overall dietary quality [11,12,23,24]. Epidemiological evidence shows that higher fruit and vegetable intake is associated with lower cardiovascular risk, lower all-cause mortality, and more favorable population health outcomes [7,8,9,11].

The Global Burden of Disease dietary risk assessment offers another important argument for linking healthy dietary behavior with population health outcomes. The GBD 2017 Diet Collaborators [11] demonstrated that suboptimal diet contributes substantially to deaths and disability-adjusted life years worldwide, with low fruit intake among the leading dietary risks. Although the GBD framework covers a broader set of dietary components than those included in the present dataset, it clearly highlights the public health relevance of diet as a modifiable determinant of chronic disease [11,21]. Moreover, dietary exposure is shaped by socioeconomic development, food systems, and policy environments, suggesting that differences in dietary behavior across countries cannot be fully understood without considering broader economic, institutional, and cultural contexts [4,5,6,11,13]. This view is also consistent with research emphasizing that food consumption and cardiovascular risk must be addressed through solutions that focus not only on individual choices but also on the globalized food system and the social environments in which diets are formed [12,13].

Several mechanisms may explain why fruit and vegetable intake is associated with life expectancy and healthy life years. First, higher consumption of fruits and vegetables may contribute to cardiovascular protection by favorably affecting blood pressure, lipid metabolism, endothelial function, and inflammatory status [7,12,13]. Second, plant-rich diets may reduce the risk of obesity and metabolic disease by lowering energy density, increasing fiber intake, enhancing satiety, and improving glycemic regulation [20,23,24]. Third, fruit and vegetable consumption often falls within a broader behavioral profile characterized by greater health awareness, a stronger preventive orientation, higher educational attainment, and greater adherence to public health recommendations [4,5,6,24]. In this sense, daily fruit and vegetable intake can serve as both a nutritional indicator and a marker of broader healthy lifestyle patterns [7,8,9].

However, the relationship between healthy dietary behavior and population health outcomes should be interpreted with caution in a cross-sectional country-level analysis. Ecological data cannot identify individual-level causal mechanisms, and national averages may conceal substantial inequalities within countries [4,5,6]. Cultural meanings, survey response patterns, and food classification practices may also influence how dietary behaviors are reported and compared across societies [1,2]. In addition, life expectancy and healthy life years reflect the cumulative influence of many factors beyond current diet, including healthcare quality, income distribution, education, occupational risks, environmental exposures, and long-term behavioral histories [4,5,6,21]. These limitations do not diminish the analysis’s relevance. Still, they indicate that the association between diet and population health should be understood within a broader systems perspective rather than as a direct, isolated effect [1,2,11,13].

Recent evidence also suggests that dietary quality should not be evaluated solely through fruit and vegetable consumption. Healthy dietary behavior should be conceptualized as a multidimensional construct encompassing meal structure, breakfast consumption, late-night eating habits, beverage choices, and sleep-related behavioral patterns. These dimensions may interact with sleep and physical activity in shaping body composition, cardiovascular risk, metabolic health, and long-term health outcomes. Sulis et al. [22] showed that nutritional and sleeping habits are interrelated with somatic health indicators in young adults, including body composition and blood pressure. Therefore, in the present study, daily fruit and vegetable consumption is interpreted as a harmonized country-level proxy for healthy dietary behavior, rather than as a comprehensive measure of dietary quality.

Countries with higher daily fruit and vegetable consumption are projected to have more favorable population health outcomes, including longer life expectancy and healthier years of life, according to the literature. On this basis, the following hypothesis is put forward:

**Hypothesis** **H1.** *Healthy dietary behavior is positively associated with population health outcomes in EU countries.*

### 2.2. Health-Enhancing Physical Activity, Life Expectancy, and Excess Weight

Physical activity is a major modifiable component of population health, being associated with lower mortality, longer life expectancy, better functional capacity, and reduced risk of non-communicable diseases [14,15,16,17,18,25]. Its effects are linked to cardiovascular fitness, insulin sensitivity, blood pressure regulation, lipid metabolism, musculoskeletal functioning, and mental health. At the same time, physical activity is not only an individual habit; working conditions, transport systems, urban planning, access to sports infrastructure, income, education, and cultural norms also shape it. Therefore, differences in physical activity across EU countries should be interpreted in the context of broader lifestyle and social environments.

The relationship between physical activity and excess weight is also important, but it should be understood as complex rather than mechanical. At the individual level, body weight reflects the interaction between energy expenditure, dietary intake, metabolism, sleep, stress, age, sex, genetic predisposition, medication use, and the broader social environment. At the population level, low physical activity may coexist with sedentary work, motorized transport, urban environments that discourage walking, and food systems that promote excessive energy intake. Therefore, countries with higher levels of health-enhancing physical activity may be expected to show lower levels of overweight and obesity. Still, this association may be weaker than anticipated when dietary habits, socioeconomic inequalities, and other contextual factors are taken into account. This interpretation is consistent with the broader obesity literature, which treats excess weight as a systems-level public health outcome shaped by behavioral, social, economic, and environmental conditions [19,20].

The distinction between healthy dietary behavior and health-enhancing physical activity is particularly relevant for the present study. Although both behaviors support health, they do not necessarily form a single empirical construct at the country level. Some countries may report relatively favorable fruit and vegetable consumption but only moderate physical activity, while others may show stronger physical activity profiles without equally favorable dietary indicators. For this reason, the present study treats physical activity as an independent predictor rather than as a component of a broader latent lifestyle construct. This approach is also more appropriate for an exploratory comparative analysis based on a limited number of country-level observations, where the purpose is not to impose a rigid causal model, but to identify differentiated lifestyle–health patterns across EU Member States.

The relationship between physical activity and population health should also be interpreted from a life-course perspective. Regular physical activity is associated not only with longer survival but also with healthier aging, preserved mobility, lower frailty, and better quality of life [15,16,17,18,25]. Healthy life years may therefore be a particularly relevant outcome, because this indicator reflects not only the length of life, but also the extent to which additional years are lived in good health. At the same time, healthy life years may be influenced by disability reporting, social expectations, access to healthcare, and national differences in self-reported health. Consequently, the association between physical activity and healthy life years may be less direct than that with life expectancy, especially in a cross-sectional, country-level analysis.

In this research, health-enhancing physical activity is expected to be positively associated with life expectancy and negatively associated with excess weight prevalence. Therefore, the following hypothesis is suggested:

**Hypothesis** **H2.** *Health-enhancing physical activity is positively associated with life expectancy and negatively associated with excess weight prevalence.*

### 2.3. Excess Weight and Population Health Outcomes

Overweight and obesity are major public health challenges in Europe and worldwide. Their prevalence has increased substantially in recent decades, and obesity is now widely understood as a complex condition shaped by biological vulnerability, dietary behavior, physical inactivity, socioeconomic inequalities, food systems, urban environments, commercial determinants, and policy contexts [19,20]. From this perspective, excess weight should be interpreted both as a biomedical risk and as a population-level outcome of social, economic, and environmental systems.

Clinical and epidemiological evidence also indicates that nutritional status can influence the progression and management of obesity-related metabolic disorders [11,20,21,24,26,27,28,29]. Inadequate diet quality and excess adiposity may aggravate cardiometabolic risk, insulin resistance, chronic inflammation, and functional limitations, whereas nutrition-oriented interventions, especially when combined with physical activity, can support weight control and reduce metabolic risk [17,18,21,25,26,29]. Although the present study does not analyze individual clinical trajectories, these mechanisms provide the clinical rationale for treating dietary behavior, excess weight, and lifestyle risks as interconnected population-level indicators [20,21,29].

The adverse health consequences of excess weight are well documented. High body mass index is associated with an increased risk of cardiovascular disease, type 2 diabetes, hypertension, dyslipidemia, several forms of cancer, musculoskeletal disorders, reduced mobility, poorer self-rated health, and premature mortality [21]. These risks are particularly relevant to comparative public health analysis because excess weight may influence both mortality and morbidity and increase pressure on healthcare systems and social protection structures. Large-scale epidemiological evidence has also shown that excess body weight is associated with substantial health losses across countries and over time [26]. In addition, collaborative analyses of BMI and cause-specific mortality have confirmed that higher BMI categories are associated with increased mortality risk. However, the magnitude of the relationship varies by population and cause of death [27]. Flegal et al. [28] further showed that obesity is associated with higher all-cause mortality, while also emphasizing that the relationship varies across BMI categories. The World Health Organization European Regional Obesity Report has also documented the links between obesity, disease burden, healthcare costs, disability, stigma, and social inequality [29]. These findings justify including excess weight as a core indicator in a systems-oriented analysis of lifestyle and population health.

In the present study, excess weight is assessed primarily through overweight prevalence, while obesity prevalence is used as a robustness indicator. This distinction is important because overweight and obesity may not relate to health outcomes in the same way. Overweight captures a broader segment of the population and may indicate early or intermediate metabolic risk. In contrast, obesity reflects a more severe form of excess weight and may be more strongly associated with illness, disability, and mortality [26,27,28,29]. At the EU level, however, using overweight and obesity simultaneously in the same regression model would be problematic because the two indicators are conceptually close and statistically overlapping. For this reason, overweight prevalence is used as the primary indicator of excess weight. In contrast, obesity prevalence is retained to assess whether the results remain consistent for a more severe weight category.

At the same time, BMI-based indicators have important limitations because they do not directly measure body composition, fat distribution, or metabolically harmful adiposity. Recent body-composition research has shown that normal BMI may coexist with excessive body fat and less favorable cardiometabolic profiles, a phenomenon often described as normal-weight obesity. For example, Falbová et al. [30] showed that normal-weight obesity in young adults is related to body-composition parameters and lifestyle habits. This evidence suggests that overweight and obesity prevalence should be interpreted as conventional anthropometric indicators rather than as complete measures of adiposity-related health risk. However, body fat percentage, waist circumference, visceral adiposity, and other body-composition parameters could not be included in the present study because comparable EU-level indicators were not available.

Healthy life years may be more sensitive than life expectancy to the burden of overweight and obesity, because excess body weight often affects morbidity, functional limitations, disability, and perceived health before it is reflected in mortality. Obesity can reduce the number of years lived in good health through several pathways, including diabetes, cardiovascular complications, osteoarthritis, reduced mobility, and poorer mental well-being [26,29]. Therefore, even when the association between overweight prevalence and life expectancy is moderate, excess weight may remain relevant for understanding morbidity-related outcomes and quality of life at the population level.

From a systems perspective, excess weight should be interpreted both as a health risk and as a marker of broader lifestyle environments. Countries with higher levels of excess weight may also display less favorable configurations of diet, physical activity, socioeconomic inequality, food availability, and health literacy. For this reason, the present study does not treat overweight only as an isolated predictor of health outcomes, but also as part of wider lifestyle–health profiles. This interpretation is consistent with the idea that obesity and overweight emerge from the interaction of behavioral, social, economic, and institutional conditions, rather than from individual choices alone [20,29].

Building on this knowledge, countries with a higher prevalence of excess weight are expected to have less favorable population health outcomes, particularly lower life expectancy and healthy life years. However, this association may be attenuated at the ecological level because EU Member States differ substantially in healthcare capacity, socioeconomic conditions, dietary environments, behavioral histories, prevention policies, and other contextual factors. The following hypothesis is therefore interpreted cautiously:

**Hypothesis** **H3.** *Excess weight prevalence is negatively associated with population health outcomes, particularly life expectancy.*

### 2.4. Daily Smoking, Healthy Dietary Behavior, and Population Health Outcomes

Smoking remains one of the most harmful behavioral risk factors for population health, contributing to cardiovascular disease, respiratory disease, cancer, and premature mortality [31]. However, in cross-sectional country-level analyses, the association between current smoking prevalence and life expectancy may appear weaker than expected because smoking-related mortality often reflects exposures accumulated over several decades [31,32]. National differences in healthcare systems, socioeconomic development, occupational exposures, alcohol consumption, obesity, and environmental conditions may further influence this relationship. Therefore, daily smoking is interpreted here both as a health risk and as a marker of broader lifestyle–health profiles.

Smoking may also be relevant as a marker of behavioral clustering. Higher smoking prevalence may coexist with less favorable dietary habits, lower health literacy, weaker preventive orientation, and greater exposure to socioeconomic disadvantage. Previous research on health behaviors has shown that lifestyle risks tend to cluster, while health-promoting behaviors tend to co-occur [33,34]. In this sense, smoking may be negatively associated with healthy dietary behavior, not because tobacco use directly reduces fruit and vegetable consumption, but because both behaviors may reflect broader social, cultural, and psychological patterns. This interpretation is particularly useful in comparative public health research, where national averages capture lifestyle configurations rather than individual behavioral mechanisms.

Smoking also contributes to health inequalities at the European level. Research on socioeconomic differences in health has shown that health behaviors are important mechanisms by which social inequalities translate into unequal health outcomes [5,6]. Petrovic et al. [35] further emphasized that behaviors such as smoking, diet, alcohol consumption, and physical activity contribute to socioeconomic inequalities in health. Since smoking often remains more prevalent among socially disadvantaged groups, its population-level impact may be intertwined with education, income, employment conditions, and access to preventive healthcare [35]. Daily smoking should therefore be interpreted both as a direct health risk and as an indicator of less favorable lifestyle–health profiles.

Based on this literature, daily smoking is projected to be negatively connected with good dietary behavior and, to a lesser extent, community health outcomes. Based on this, the following hypothesis is proposed:

**Hypothesis** **H4.** *Daily smoking is negatively associated with healthy dietary behavior and population health outcomes.*

### 2.5. Sugar-Sweetened Soft Drink Consumption and Lifestyle–Health Profiles

Sugar-sweetened soft drink consumption is relevant for nutrition and public health because these beverages provide rapidly absorbable sugars, increase energy intake, and have low nutritional value. Previous studies have associated sugar-sweetened beverages with weight gain, obesity, type 2 diabetes, metabolic syndrome, cardiovascular risk, and poorer diet quality [36,37,38,39,40]. In a cross-sectional country-level analysis, however, direct associations with population health outcomes may be difficult to detect because consumption patterns are shaped by age structure, income, marketing, food prices, cultural preferences, and other contextual factors.

However, the direct association between sugar-sweetened soft drink consumption and population health outcomes may be difficult to identify in a cross-sectional country-level analysis. Consumption patterns can be shaped by age structure, income, marketing, food prices, cultural preferences, urbanization, and generational habits. In some countries, higher soft drink consumption may coexist with stronger healthcare systems, higher socioeconomic resources, or other protective conditions, which can obscure direct associations with life expectancy or healthy life years. For this reason, the present study interprets this variable not only as a possible direct predictor of health outcomes but also as an indicator that may help describe broader lifestyle–health profiles.

Sugar-sweetened soft drink consumption is particularly relevant for typological analysis. Countries with higher consumption may be grouped with other less favorable indicators, such as weaker healthy dietary behavior, lower physical activity, higher smoking prevalence, or higher excess weight. Even when bivariate correlations are weak, the variable may still help identify behavioral configurations across countries. This interpretation is consistent with the logic of cluster analysis, which focuses on indicator combinations rather than isolated linear relationships. It also fits the broader view that lifestyle risks often cluster, while health-promoting behaviors may cluster as well [33,34].

From a policy perspective, this indicator remains relevant because sugar-sweetened beverage consumption can be addressed through taxation, marketing restrictions, school food regulations, labeling, and public education [41,42].

Based on this literature, sugar-sweetened soft drink consumption is expected to be associated with less favorable lifestyle and health profiles across EU countries. However, this relationship may not appear as a strong direct bivariate association at the ecological level, because countries differ in age structure, socioeconomic resources, food markets, cultural habits, healthcare systems, and broader lifestyle configurations. Thus, the following hypothesis is interpreted mainly in terms of lifestyle–health profiles rather than as a simple direct association:

**Hypothesis** **H5.** *Sugar-sweetened soft drink consumption is associated with less favorable lifestyle and health profiles across EU countries.*

### 2.6. Lifestyle–Health Typologies in the European Union

The relationships among diet, physical activity, smoking, sugar-sweetened soft drink consumption, excess weight, and population health outcomes are unlikely to follow the same pattern across all EU countries. Member States differ in economic development, social protection, education, food systems, healthcare capacity, prevention policies, cultural habits, and historical exposure to behavioral risks [4,5,6,22]. For this reason, a systems-oriented approach should not be limited to testing whether one variable predicts another. It should also examine how several behavioral and health indicators combine into broader national profiles. Such an approach is particularly relevant in public health research, where population outcomes often reflect the cumulative influence of multiple behaviors, risks, and contextual conditions rather than the isolated effect of a single factor [1,2,3,21].

Cluster analysis offers a useful framework for comparative public health research by grouping countries based on similarities across several indicators, rather than assuming that all Member States follow the same lifestyle–health pathway. This method is appropriate when relationships between variables are heterogeneous, nonlinear, or context-dependent, as is often the case in population health research. European evidence also supports this analytical direction, since smoking, obesity, alcohol consumption, and other lifestyle-related risks have influenced life-expectancy trends differently across countries, genders, and regions [22].

From a policy perspective, lifestyle–health typologies can support more differentiated public health interventions. Countries with weaker dietary behavior, higher smoking, higher excess weight, or higher sugar-sweetened soft drink consumption may require different combinations of nutrition education, tobacco control, obesity prevention, food-environment regulation, labeling, taxation, or school-based interventions [41,42]. In this way, typologies can translate statistical patterns into more targeted policy insights.

A cluster-based approach is also consistent with the article’s systems perspective. Population health outcomes emerge from the convergence of multiple determinants, not from a single dominant behavioral variable. By grouping countries based on multiple lifestyle and health indicators, the analysis can reveal patterns that are more realistic than those from simple linear models. This is particularly appropriate for the present dataset, which includes only 27 EU Member States and therefore requires parsimonious modeling, cautious interpretation, and attention to broader national configurations.

Based on this reasoning, EU countries are expected to form distinct lifestyle–health typologies. Accordingly, the following hypothesis is proposed:

**Hypothesis** **H6.** *EU countries can be grouped into distinct lifestyle–health typologies based on dietary behavior, physical activity, excess weight, lifestyle risks, and population health outcomes.*

## 3. Materials and Methods

### 3.1. Research Design

The research design of this study is cross-sectional, comparative, and exploratory, encompassing the 27 Member States of the European Union. The research does not attempt to establish causal effects, but rather to identify statistically significant associations between lifestyle behaviors, excess weight, and population health outcomes at the country level. This method is appropriate because the data concern aggregated national variables rather than individual respondents, and because the number of observations is limited to the EU-27. Thus, the empirical strategy implies parsimonious statistical testing, interpretation, and triangulation using numerous complementary methodologies.

The limited number of observations is an important methodological constraint. Since the unit of analysis is the EU Member State, the sample includes only 27 countries, reducing statistical power and limiting the ability to estimate complex multivariable models. For this reason, the empirical analysis is interpreted as exploratory and comparative rather than confirmatory. The results are assessed mainly by the direction, magnitude, and consistency of associations across complementary methods, not by isolated significance tests.

The study adopts a systems-oriented reasoning. Instead of treating healthy eating, physical activity, smoking, sugar-sweetened soft drink use, overweight, and health outcomes as separate variables, it evaluates how these indicators interact with one another and how they build into broader lifestyle–health profiles. This approach aligns with the view that population health is an emergent property of numerous behavioral and contextual variables that interact unevenly across countries. The study design thus combines typological analysis and association testing.

The empirical model is organized around six hypotheses. The first hypothesis tested whether community health outcomes were positively connected with healthy eating behavior, defined by daily fruit and vegetable consumption. The second hypothesis tests the positive association between health-enhancing physical exercise and life expectancy and the negative association with excess weight. The third hypothesis concerns the unfavorable association between excess weight and health outcomes. The fourth hypothesis looks at the link between daily smoking and poor eating behavior and lower health consequences. The fifth hypothesis posits that sugar-sweetened soft drink intake is a marker of a less healthy lifestyle and health characteristics. The sixth hypothesis examines whether EU countries can be categorized into distinct lifestyle–health typologies. The design thus embraces both explanatory and classificatory goals, enabling the study to uncover both individual associations and broader national patterns.

### 3.2. Selected Data

The empirical analysis uses cross-sectional data from 2019 for 27 European Union Member States. The primary source is the European Health Interview Survey (EHIS), especially wave 3, conducted in 2019 across EU Member States using a harmonized Eurostat methodological framework, complemented by relevant health measures from Eurostat [43]. The EHIS wave 3 methodological manual provides conceptual guidelines, a model questionnaire, translation instructions, and statistical survey recommendations to ensure comparability of health, lifestyle, and healthcare indicators across European countries [43]. The EHIS data are appropriate for this investigation, as they include harmonized information on health habits, body weight, and self-reported health across European countries. This harmonization enables comparison and avoids inconsistencies that often arise from national surveys that use different definitions, age groups, or measurement techniques.

The dataset’s metrics are organized into four analysis domains. The first domain, healthy eating behavior, covers daily fruit and vegetable intake. These two variables are used to construct a composite indicator of healthy eating behavior because they represent closely linked characteristics of food quality that are widely used in public health surveillance. The empirical results showed that these two indicators had adequate internal consistency for a two-item composite measure and may be aggregated into a single dietary score.

This operationalization is justified by the harmonized availability of daily fruit and vegetable consumption indicators in EHIS and by their frequent use in public health monitoring. However, the indicator should not be interpreted as a complete measure of dietary quality. Dietary traditions differ substantially across EU countries, and overall diet quality may also depend on meal composition, food processing, total energy intake, added sugars, salt, fats, fiber, whole grains, legumes, nuts, fish, and broader dietary patterns [11,12,23,24]. Therefore, HDB is used in this study as a comparable country-level proxy for healthy dietary behavior, not as a comprehensive measure of national diet quality.

The second domain is health-enhancing physical activity. This variable is considered separately from dietary behavior, not as part of the same composite indicator. Theoretically and empirically, this decision is justified. A healthy lifestyle includes both diet and physical activity, but these may differ across the nation. Some countries may have high fruit and vegetable consumption but low physical activity. Others may have more robust physical activity profiles, but less beneficial dietary indicators. Therefore, incorporating physical activity as a separate variable allows for a more nuanced interpretation at the country level.

The third area consists of lifestyle risk markers and excess weight. Daily smoking and sugar-sweetened soft drink drinking are behavioral risk markers. The basic measure of excess weight is prevalence. Obesity prevalence is retained for robustness checks and supplemental interpretation. Overweight and obesity are not included in the same regression model, as they are conceptually and statistically related. Using them together is prone to redundancy and multicollinearity. Instead, the prevalence of overweight indicates a larger population-level burden of excess weight, whereas the prevalence of obesity reflects a more severe weight category.

The fourth domain is the population’s health outcomes. The primary outcome is life expectancy at birth, a robust and comparable measure of population health performance. Healthy life years are used as a secondary outcome because they measure not only survival but also quality of life. Together, these characteristics enable the analysis to connect lifestyle choices not just to longevity but also to broader health circumstances.

It should be noted that the dietary variables used in this study capture intake-related behaviors, not clinical nutritional status or biomarker-based nutrient profiles. Therefore, daily fruit and vegetable consumption and sugar-sweetened soft drink intake are interpreted as population-level dietary indicators rather than direct measures of micronutrient status, metabolic regulation, or disease-specific nutritional changes. This distinction is important because dynamic changes in essential nutrients across obesity, type 2 diabetes, hypertension, and other metabolic disease states may be clinically relevant but cannot be directly assessed using the harmonized country-level indicators used in the present study.

The final dataset, therefore, comprises national-level indicators (Table 1).

Since the unit of analysis is the country, all findings refer to associations between national profiles and should not be interpreted as individual-level behavioral effects.

### 3.3. Methods

The empirical analysis was organized into consecutive phases, reflecting the comparative-exploratory nature of the study. Given that the research relies on country-level observations for 27 European Union Member States, the methodological strategy was intentionally modest. It included descriptive analysis, composite indicator generation, bivariate correlation analysis, simplified regression modeling, and cluster analysis. This sequence enabled us to explore the proposed correlations and to uncover wider lifestyle–health combinations across countries.

First, descriptive statistics were conducted for all variables included in the study. The distribution of dietary behavior, physical activity, smoking, consumption of sugar-sweetened soft drinks, excess weight, and population health outcomes across countries was assessed using means, standard deviations, and minimum and maximum values. This preliminary step was important because interpreting country-level data depends not only on average values but also on the degree of heterogeneity among Member States.

Secondly, healthy dietary behavior was constructed as a composite index based on daily fruit and vegetable consumption. Reliability of this two-item measure was examined with Cronbach’s alpha [50], where *α* is Cronbach’s alpha for a scale with *k* items:

(1) $$\alpha =\frac{k}{k-1}\left(1-\frac{\sum_{i=1}^{k}\sigma_{i}^{2}}{\sigma_{T}^{2}}\right)$$

where $\sigma_{i}^{2}$ represents the variance of the item i, and $\sigma_{T}^{2}$ represents the variance of the total score.

After standardizing fruit and vegetable consumption, the healthy dietary behavior (HDB) indicator was computed as the arithmetic mean of the two standardized scores:

(2) $${\mathrm{HDB}}_{j}=\frac{{\mathrm{ZDFC}}_{j}+{\mathrm{ZDVC}}_{j}}{2}$$

${\mathrm{HDB}}_{j}$—healthy dietary behavior in the country j;
${\mathrm{ZDFC}}_{j}$—standardized daily fruit consumption;
${\mathrm{ZDVC}}_{j}$—standardized daily vegetable consumption.

Third, Pearson and Spearman correlation coefficients were used to test the bivariate associations linked to the hypotheses. Pearson correlation measured the strength of linear relationships between two variables:

(3) $$r_{xy}=\frac{\sum_{j=1}^{n}\left(x_{j}-\bar{x}\right)\left(y_{j}-\bar{y}\right)}{\sqrt{\sum_{j=1}^{n}{\left(x_{j}-\bar{x}\right)}^{2}\sum_{j=1}^{n}{\left(y_{j}-\bar{y}\right)}^{2}}}$$

$r_{xy}$—the Pearson correlation coefficient between the variables *x* and *y*;
$x_{j}$—the value of variable x for observation or statistical unit *j*;
$y_{j}$—the value of variable y for the same observation *j*.

Since the sample contains only 27 countries and some indicators may be affected by outliers or non-normal distributions, Spearman’s rank correlation was also used as a robustness measure [50]:

(4) $$\rho =1-\frac{6\sum d_{j}^{2}}{n\left(n^{2}-1\right)}$$

$d_{j}$—the difference between the ranks of the two variables for the country j;
n—the number of countries.

Because several bivariate associations and reduced regression models were examined, the possibility of multiplicity was considered when interpreting the results. No formal correction for multiple testing was applied because the study is exploratory, hypothesis-guided, and based on a small number of country-level observations. Applying a strict correction would further reduce statistical power. Therefore, statistical significance is interpreted cautiously, along with effect size, theoretical plausibility, consistency between Pearson and Spearman coefficients, regression results, and cluster-based patterns.

Fourth, reduced linear regression models were estimated. Given the limited number of observations, only a few theoretically relevant predictors were included. The main model estimated life expectancy as a function of healthy dietary behavior, health-enhancing physical activity, and overweight prevalence:

(5) $${\mathrm{LEB}}_{j}=\beta_{0}+\beta_{1}{\mathrm{HDB}}_{j}+\beta_{2}{\mathrm{HEPA}}_{j}+\beta_{3}{\mathrm{BMIOV}}_{j}+\varepsilon_{j}$$

A similar model was estimated for healthy life years:

(6) $${\mathrm{HLY}}_{j}=\beta_{0}+\beta_{1}{\mathrm{HDB}}_{j}+\beta_{2}{\mathrm{HEPA}}_{j}+\beta_{3}{\mathrm{BMIOV}}_{j}+\varepsilon_{j}$$

${\mathrm{LEB}}_{j}$—life expectancy at birth in the country j.
$HLY_{j}$—healthy life years in the country j.
$HDB_{j}$—healthy dietary behavior in the country j.
$HEPA_{j}$—health-enhancing physical activity in the country j.
$BMIOV_{j}$—the prevalence of overweight in the country j.
$\beta_{0}$—the intercept.
$\beta_{1}$, $\beta_{2}$, and $\beta_{3}$—the estimated regression coefficients for healthy dietary behavior, health-enhancing physical activity, and overweight prevalence, respectively.$\varepsilon_{j}$—the random error associated with the country j.

Additional models included daily smoking and sugar-sweetened soft drink consumption as supplementary predictors. Still, these results were interpreted cautiously because larger models may become unstable in a small country-level sample.

Finally, cluster analysis was used to identify lifestyle–health typologies among EU Member States. All clustering variables were standardized before analysis. Hierarchical clustering was conducted using Ward’s method and squared Euclidean distance:

(7) $$d_{ij}^{2}=\sum_{p=1}^{m}{\left(z_{ip}-z_{jp}\right)}^{2}$$

$d_{ij}^{2}$—the distance between countries i and j;
$z_{ip}$ and $z_{jp}$—standardized values for the variable p.

Ward’s method was selected because it minimizes within-cluster variance and generally produces compact, interpretable clusters [51]. K-means clustering was then applied as a complementary procedure. This method minimizes the total within-cluster sum of squares:

(8) $$min\sum_{c=1}^{K}\sum_{j\in C_{c}}{∥x_{j}-\mu_{c}∥}^{2}$$

$C_{c}$—cluster c;
$x_{j}$—the vector of standardized indicators for the country j;
$\mu_{c}$—the centroid of the cluster c.

Differences between clusters were examined using ANOVA and the Kruskal–Wallis test. This combination of methods enabled evaluation of the hypotheses while preserving a cautious interpretation appropriate for cross-sectional, country-level data.

## 4. Results

### 4.1. Descriptive Statistics and Construction of the Healthy Dietary Behavior Indicator

The analysis was conducted on the 27 European Union Member States using country-level indicators from the 2019 European Health Interview Survey and related Eurostat health data. The descriptive statistics indicate substantial heterogeneity across countries in both lifestyle behaviors and population health outcomes (Table 2).

Daily fruit consumption ranged from 23.7% to 69.5%, and daily vegetable consumption ranged from 17.4% to 78.4%. For health-enhancing physical exercise, the range was much larger, from 9.9% to 95.4%. The frequency of overweight ranged from 44.7% to 63.9%, and life expectancy ranged from 75.1 to 84.0 years.

Healthy dietary behavior was calculated as the mean of standardized scores for daily fruit and vegetable consumption. Because the indicator is based on standardized scores, its mean is approximately zero by construction. Therefore, the value of 0.00 reported in Table 2 should not be interpreted as the absence of healthy dietary behavior, but rather as the reference point around which EU countries vary relative to it. The two dietary indicators demonstrated acceptable internal consistency for the two-item composite measure (Cronbach’s alpha = 0.706). Both Pearson and Spearman correlations showed a positive, substantial association between daily fruit and daily vegetable consumption, which justifies aggregating the two variables into a single indicator of dietary behavior.

### 4.2. Bivariate Associations Between Lifestyle Behaviors and Health Outcomes

Pearson and Spearman correlation coefficients were used to examine the main associations corresponding to the six hypotheses. Given the small number of observations, Spearman’s rank correlation coefficients were also considered important because they are less sensitive to outliers and non-normal distributions (Table 3).

Healthy dietary behavior was positively and significantly associated with life expectancy in the Pearson correlation analysis (r = 0.459, *p* = 0.016). The Spearman coefficient was also positive, although only marginally significant (rho = 0.361, *p* = 0.064). The associations with healthy life years were positive but not statistically significant. These results offer partial support for H1, suggesting that healthier dietary behavior is more clearly related to life expectancy than to the other health outcomes.

Health-enhancing physical activity was positively associated with life expectancy (r = 0.435, *p* = 0.023), but its association with healthy life years was negligible. The relationship between physical activity and overweight prevalence was negative but not statistically significant; therefore, it is not interpreted as evidence of an association in this dataset (r = −0.320, *p* = 0.103). No meaningful association was found between physical activity and obesity prevalence. Therefore, H2 receives partial empirical support, mainly through the positive relationship between physical activity and life expectancy.

Overweight prevalence was negatively and significantly associated with life expectancy (r = −0.505, *p* = 0.007; rho = −0.568, *p* = 0.002). Its association with healthy life years was also negative, but weaker in the Pearson analysis and only marginally significant in the Spearman analysis. When obesity prevalence was used as a robustness indicator, the strongest relationship appeared with healthy life years in the Spearman correlation (rho = −0.449, *p* = 0.019). These results support H3 mainly for life expectancy and suggest that obesity may be more relevant for morbidity-related outcomes than for life expectancy alone.

Daily smoking was strongly and negatively associated with healthy dietary behavior (r = −0.572, *p* = 0.002; rho = −0.597, *p* = 0.001). However, its direct associations with life expectancy and healthy life years were weak and statistically nonsignificant. These findings partially support H4. They suggest that smoking is more closely associated with behavioral clustering than with immediate country-level health outcomes in this cross-sectional dataset.

Sugar-sweetened soft drink consumption did not show the expected adverse associations with excess weight or health outcomes. Its correlations with overweight prevalence and life expectancy were close to zero, whereas its association with healthy life years was positive rather than negative, although not significant at the 0.05 level. Therefore, H5 is not supported as a direct bivariate relationship. This variable was retained mainly for the cluster analysis, where it may still contribute to lifestyle–health profiles.

### 4.3. Reduced Regression Models

Given the small sample size, reduced regression models were estimated with only a few predictors.

In the basic regression models, healthy dietary behavior explained 21.1% of the difference in life expectancy (R^2^ = 0.211, *p* = 0.016), and health-boosting physical activity explained 18.9% (R^2^ = 0.189, *p* = 0.023). Overweight explained 25.5% of the variance in life expectancy (R^2^ = 0.255, *p* = 0.007), highlighting the importance of excess weight as a population-level health indicator. Daily smoking accounted for 32.8% of the variation in healthy dietary behavior (R^2^ = 0.328, *p* = 0.002), supporting the behavioral clustering hypothesis.

Then, a simplified multiple regression model was estimated for life expectancy, with good dietary behavior, health-enhancing physical activity, and overweight prevalence as predictors (Table 4 and Table 5).

The model was statistically significant and explained 47.4% of the variance in life expectancy (R^2^ = 0.474, adjusted R^2^ = 0.405, *p* = 0.002). Healthy dietary behavior remained a positive predictor (β = 0.382, *p* = 0.023), while health-enhancing physical activity also had a beneficial effect (β = 0.339, *p* = 0.046), and the prevalence of overweight had a negative, though marginally significant, coefficient (β = −0.298, *p* = 0.085). Adding daily smoking and sugar-sweetened soft drink use marginally increased the model’s explanatory power (R^2^ = 0.479), but neither was a significant contributor. This result explains their more conservative interpretation as profile indicators rather than strong direct predictors of longevity.

The reduced regression models were not statistically significant for healthy years of life. This result indicates that HLY may be affected by other factors not included in the present dataset, such as disability patterns, access to healthcare, social protection, reporting differences, and broader socioeconomic factors.

Overall, these findings indicate that the regression models explain only part of the variation in population health outcomes. The model for life expectancy has moderate explanatory power, whereas the models for healthy life years explain a limited proportion of variance and are not statistically significant. Therefore, the regression results should be interpreted as exploratory evidence of selected country-level associations rather than as comprehensive explanatory models of population health.

### 4.4. Lifestyle–Health Typologies of EU Countries

**Figure 1:**
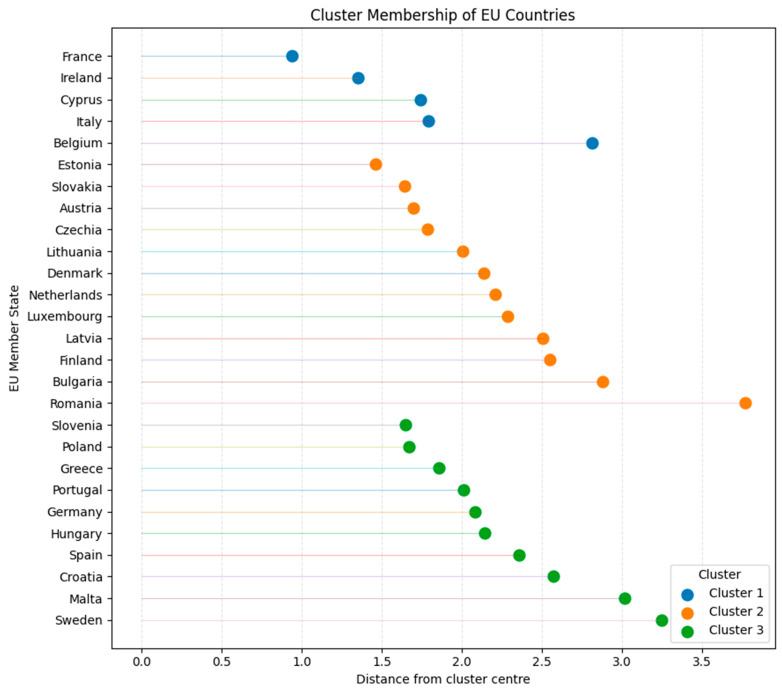
Cluster membership of EU countries. Note: Cluster 1—Healthy-diet, low-excess-weight and high-longevity profile; Cluster 2—Lower-diet-quality, higher-smoking and lower-health-outcome profile; Cluster 3—Mixed lifestyle–health profile with low smoking and higher excess weight.

To examine H6, the study used a two-step clustering strategy. First, hierarchical cluster analysis was performed using Ward’s method and squared Euclidean distance to explore the underlying grouping structure among EU countries. Second, K-means clustering was applied with a predefined three-cluster solution, using standardized values for daily fruit consumption, daily vegetable consumption, health-enhancing physical activity, daily smoking, sugar-sweetened soft drink consumption, overweight prevalence, healthy life years, and life expectancy (Figure 1).

The three-cluster solution was retained because it provided the most interpretable classification of EU Member States and differentiated countries along meaningful lifestyle–health configurations.

The K-means procedure converged after four iterations, indicating a stable solution. The final cluster centers show that the three groups differ mainly in dietary behavior, smoking, overweight prevalence, and health outcomes, while health-enhancing physical activity contributes less clearly to the separation of countries. The distances between the final cluster centers also indicate that Cluster 1 is more clearly separated from Cluster 2, whereas Cluster 3 occupies an intermediate position between the two (Table 6).

Cluster 1 includes Belgium, Ireland, France, Italy, and Cyprus. This group exhibits the most favorable dietary profile, with the highest standardized values for daily fruit and vegetable consumption. It is also characterized by the lowest prevalence of overweight, above-average life expectancy, and healthy life years. Daily smoking is below the EU average in this cluster, while sugar-sweetened soft drink consumption is above average. Despite this latter aspect, the overall profile suggests a relatively favorable lifestyle–health configuration, driven mainly by stronger dietary behavior, lower excess weight, and better population health outcomes. This cluster can therefore be described as a healthy diet, low excess weight, and a high longevity profile.

Cluster 2 includes Bulgaria, Czechia, Denmark, Estonia, Latvia, Lithuania, Luxembourg, the Netherlands, Austria, Romania, Slovakia, and Finland. This cluster shows the least favorable dietary pattern, with below-average fruit and vegetable consumption. It also has the highest level of daily smoking and the lowest values for life expectancy and healthy life years. Overweight prevalence is close to the EU average, while sugar-sweetened soft drink consumption is below average. Health-enhancing physical activity is slightly above the overall mean, but this does not translate into more favorable health outcomes at the cluster level. This group may therefore be interpreted as having lower diet quality, higher smoking rates, and poorer health outcomes.

Cluster 3 includes Germany, Greece, Spain, Croatia, Hungary, Malta, Poland, Portugal, Slovenia, and Sweden. This group has above-average fruit consumption, close-to-average vegetable consumption, and the lowest daily smoking rate. At the same time, it records the highest overweight prevalence among the three clusters. Life expectancy and healthy life years are above the EU average, although not as clearly favorable as in Cluster 1. This profile is therefore more mixed: it combines relatively favorable health outcomes and lower smoking with higher excess weight. It can be described as a mixed lifestyle–health profile with low smoking, moderate dietary behavior, and higher excess weight.

The ANOVA results from the K-means procedure indicate that the clusters differ most clearly in daily fruit and vegetable consumption, daily smoking, overweight prevalence, healthy life years, and life expectancy (Table 7).

There are significant differences for daily fruit consumption (F = 15.633, *p* < 0.001), daily vegetable consumption (F = 9.061, *p* = 0.001), daily smoking (F = 12.766, *p* < 0.001), overweight prevalence (F = 9.207, *p* = 0.001), healthy life years (F = 6.457, *p* = 0.006) and life expectancy (F = 4.075, *p* = 0.030). Consumption of sugar-sweetened soft drinks did not differ significantly among clusters at the 0.05 level (F = 3.142, *p* = 0.061), and health-boosting physical activity did not significantly discriminate among the three groups (F = 0.493, *p* = 0.617). These findings should be considered descriptively, as SPSS (v.27.0) rightly points out that the clusters are formed to maximize between-group differences.

The clustering results generally support H6. The different lifestyle–health typologies across EU countries can be identified, though the profiles are neither straightforward nor homogeneous. The most favorable health profile is associated with stronger eating habits and lower prevalence of overweight, rather than with increased physical activity. The least favorable profile combines poorer eating habits, more daily smoking, and poorer health results. The third profile is a more difficult scenario, with a reasonably good lifespan and healthy life years, but a higher prevalence of overweight. This reinforces the usefulness of a systems-based approach, as lifestyle and health indicators interact differently across European countries and cannot be fully understood from simple bivariate relationships in isolation.

### 4.5. Summary of Hypothesis Testing

The results provide unequal but substantial support for the proposed hypotheses. H1 is somewhat supported, since healthy dietary behavior is positively and significantly associated with life expectancy but not with healthy life years. H2 is also partly supported, as health-boosting physical activity is positively related to life expectancy and, although not substantially, is adversely related to the prevalence of overweight. H3 is mostly supported for life expectancy, since the prevalence of overweight is clearly and negatively associated with it. H4 is partially supported with significant evidence of a negative association between daily smoking and good dietary behavior, but weaker evidence of direct linkages to health outcomes. H5 is not substantiated as a direct correlation, as sugar-sweetened soft drink use is not associated with the expected detrimental relationships with excess weight or health consequences. The cluster analysis reveals unique and interpretable lifestyle–health typologies across EU countries, thereby supporting H6 (Table 8).

## 5. Discussion

The study’s results reaffirm the importance of a systems-based approach to the relationship between lifestyle habits, excess body weight, and population health in the European Union. In general, the study demonstrates that these correlations do not always follow simple, uniform, and easily recognized patterns when analyzed using linear models, especially when the country is the unit of analysis, and the number of observations is limited to the 27 Member States. Nevertheless, this non-uniformity is one of the study’s main contributions. Dietary habits, physical activity, smoking, use of sweetened beverages, excess body weight, and health outcomes are not independent variables. Rather, they blend into different national profiles, conditioned by social, cultural, economic, and institutional variables. This interpretation is consistent with complex-systems approaches to public health, which argue that population health outcomes emerge from interacting behavioral, social, environmental, and institutional determinants rather than from isolated individual risk factors [52,53]. It also aligns with recent systems-oriented obesity-prevention research, which emphasizes that obesity and related lifestyle outcomes should be addressed through interventions sensitive to behavioral, environmental, social, economic, and policy interactions [54]. World Health Organization’s position emphasizes the multidimensionality of non-communicable diseases and the need for prevention through treatments targeting modifiable behavioral factors [1,2,3]. Our findings also align with work on social determinants of health and the persistence of health inequities across European states [4,5,6].

The first hypothesis, that healthy dietary behavior is positively associated with community health outcomes, obtained partial empirical support. The composite indicator of healthy eating behavior, based on daily intake of fruits and vegetables, showed a positive, substantial correlation with life expectancy, consistent with the literature highlighting the preventive role of diets rich in plant-based foods. In meta-analyses by Wang et al. [8] and Aune et al. [9], higher intakes of fruit and vegetables were associated with a lower risk of all-cause mortality, cardiovascular disease, and certain cancers. Nevertheless, the link with healthy life years and self-perceived health was weaker. This difference indicates that life expectancy is a better measure of the cumulative effect of dietary behaviors on longevity. At the same time, healthy life years and self-perceived health also depend on factors such as disability, access to healthcare services, cultural reporting norms, and subjective expectations about health [55,56,57]. H1 can therefore be considered partly validated, especially with respect to the lifespan objective component.

The second hypothesis, that a favorable link between life expectancy and physical exercise improves health and that physical exercise is negatively associated with excess body weight, was also partially supported. The results revealed a positive relationship between HEPA and life expectancy, consistent with both traditional and modern literature on the advantages of physical activity. Physical activity is associated with a reduced risk of non-communicable diseases and premature death, consistent with early studies by Morris et al. on occupational physical activity and by Lee et al. and Ekelund et al. [14,17,18]. In contrast, the negative association between HEPA and overweight was in the predicted direction but not statistically significant. This scenario appears reasonable, as the national incidence of overweight is influenced not only by physical activity but also by caloric intake, dietary structure, urbanization, occupational sedentarism, age, income, and the broader food environment [58,59]. Thus, H2 is better supported for the association between physical activity and longevity than for a strong direct relationship between physical activity and reduced excess body weight.

The third hypothesis predicted a negative link between the prevalence of excess body weight and health outcomes, and found stronger, clearer support for this relationship with respect to life expectancy. The negative association between overweight and life expectancy indicates that countries with higher rates of excess body weight are more likely to have poorer longevity outcomes. This result is consistent with the literature, which identifies high body mass index as a key metabolic risk factor [21,26,27]. At the same time, the relationship with healthy life years was more subtle, and obesity was more relevant than general overweight for this dimension [60,61]. This finding is an important observation; clearer support for this relationship, with respect to moderate excess body weight and obesity, does not have the same health implications [62]. Flegal et al. demonstrated that the association between BMI categories and mortality was more complicated than a simple linear rise in risk across weight categories [28]. The present findings corroborate the complexity and suggest a differentiated understanding of overweight and obesity.

These findings should also be interpreted from a broader life-course perspective. Although the present study focuses on contemporary lifestyle behaviors and country-level indicators, obesity and cardiometabolic risk may also reflect developmental, reproductive, and environmental exposures accumulated over time. For example, Vorobeľová et al. [63] showed that female reproductive history and environmental factors were associated with obesity and hypertension in later life, suggesting that current weight status and cardiovascular risk cannot be explained only by present dietary behavior or physical activity. This perspective is relevant for the present study because between-country differences in life expectancy and excess weight may partly reflect long-term demographic, reproductive, social, and developmental histories that the available EHIS does not capture.

Partial evidence was found for the fourth hypothesis, that daily smoking was negatively related to good eating and health outcomes. The strongest relationship found in the investigation was the unfavorable link between daily smoking and healthy dietary habits. This finding supports the concept of clustering of risk behavior, as described in the literature on behavioral factor clustering [33,34]. In other words, smoking does not present as an isolated medical risk but as part of a less favorable behavioral profile that may also include inferior food habits. This result also has implications for public health policy. Since the present findings indicate that higher daily smoking is associated with weaker healthy dietary behavior, interventions that focus on only one behavioral domain may have limited effects. Tobacco control, nutrition education, food access policies, and broader prevention programs may therefore need to be designed as complementary components of integrated health promotion strategies. Such an approach is consistent with the view that behavioral risks are embedded in everyday routines, socioeconomic constraints, and institutional contexts, rather than being only isolated individual choices [33,34]. However, the association between smoking and health outcomes did not appear substantial in the cross-sectional data included in the study. This finding is consistent with the epidemiological research on the important impact of smoking on mortality [30,64]. Rather, it reflects the fact that the effects of smoking on life expectancy are cumulative over time and appear with a temporal delay. The smoking epidemic model suggests that the mortality impacts are seen only after some decades, making them challenging to capture using a cross-sectional indicator of current smoking [32].

At the same time, smoking-related behavior in the EU should be interpreted in light of recent changes in nicotine consumption patterns. Conventional tobacco smoking has declined in many European countries over recent decades, and adolescent smoking has decreased in most EU countries during the last decade. However, this progress has been accompanied by the increasing use of electronic cigarettes and other emerging nicotine-delivery devices, particularly among younger groups [65,66]. This shift is relevant for the interpretation of lifestyle-risk profiles because current indicators of daily smoking may not fully capture newer forms of nicotine use. Future research should therefore distinguish more clearly between conventional tobacco smoking, electronic cigarette use, heated tobacco products, and other nicotine products when assessing behavioral risks and population health outcomes.

The final hypothesis, relating sweetened beverage use to less desirable lifestyle–health profiles, was not supported through clear direct links. Daily consumption of sweetened beverages did not significantly differentiate the clusters and did not demonstrate expected detrimental relationships with excess body weight or longevity. The lack of a significant association between sugar-sweetened soft drink consumption and obesity or other health outcomes should be interpreted with caution. This result contrasts with much of the individual-level epidemiological literature, which links sugar-sweetened beverages with weight gain, obesity, type 2 diabetes, metabolic syndrome, and cardiometabolic risk [36,37,38,39,40,67]. The discrepancy may reflect the ecological and cross-sectional design of the present study. Country-level averages may conceal age, sex, socioeconomic status, and within-country differences in beverage consumption and metabolic risk. In addition, the available indicator captures one specific form of sugar-sweetened beverage consumption and does not measure total free sugar intake, portion size, frequency over the life course, substitution with other beverages, or broader dietary patterns. Stronger healthcare systems, different dietary traditions, physical activity patterns, and other contextual factors may also obscure direct associations at the country level. Therefore, the nonsignificant result should not be interpreted as evidence that sugar-sweetened beverages are unrelated to obesity or metabolic health. Rather, it indicates that this relationship was not detectable with the aggregated EU-level indicators and the limited cross-sectional sample used in this study.

The sixth hypothesis had the strongest empirical support. The cluster analysis identified three distinct typologies of EU Member States based on observed combinations of lifestyle and health indicators. The first cluster is characterized by higher fruit and vegetable intake, lower excess body weight, and higher life expectancy and healthy life years. The second cluster is characterized by lower fruit and vegetable intake, higher smoking prevalence, and lower values of population health outcomes. The third cluster shows a mixed configuration, with lower smoking prevalence and relatively higher health outcome indicators, but also higher excess body weight. These patterns should be interpreted as descriptive country-level associations, not as evidence that one behavioral factor directly causes better or worse health outcomes. The findings are consistent with Janssen et al. [22], who showed that smoking, obesity, and alcohol affected variations in life expectancy across Europe in diverse ways by area, gender, and behavioral history. The study thus supports a systems-oriented interpretation and suggests that public health strategies should consider observed national configurations rather than relying only on average associations at the European level.

The cluster interpretation should nevertheless be contextualized carefully. The three typologies identify descriptive lifestyle–health profiles, but they do not fully explain why countries belong to one group rather than another. Socioeconomic inequalities, educational attainment, income distribution, health system capacity, preventive care, social protection, and food environments may all shape the observed profiles. For example, countries with similar behavioral indicators may still differ in life expectancy or healthy life years because healthcare access, prevention policies, health literacy, and social inequalities may mitigate or amplify lifestyle-related risks. Therefore, the clusters should be interpreted as empirically useful descriptive configurations rather than as complete explanatory models of population health.

The study supports a comprehensive understanding of the link between lifestyle and population health. Healthy food and physical activity are important, but their consequences vary depending on the situation. Excess body weight remains a significant indicator of health vulnerability, but it alone does not account for cross-country variation. Smoking is a more salient measure of a negative behavioral profile than a direct cross-sectional predictor of longevity. More extensive analysis of sweetened beverages, ideally with person-level data or more precise dietary indicators, is required. The study’s most important contribution is the discovery of national typologies, confirming that population health results from complex configurations of behaviors, hazards, and social situations. This perspective may promote more varied policies for nutrition, obesity prevention, and health promotion in the European Union.

### 5.1. Theoretical Implications

This paper seeks to contribute to research on nutrition, lifestyle, and population health by offering a systems-oriented analysis of the links between dietary habits, physical activity, risk factors, excess body weight, and health outcomes across the European Union. The results demonstrate that these correlations are nonlinear, i.e., a single healthy habit does not universally and directly lead to better health outcomes. Instead, they believe that population health is a product of complex national constellations in which food, physical activity, smoking, sweetened beverage consumption, and the prevalence of excess body weight interact in diverse ways across countries. From this perspective, the study supports the view that the analysis of health behaviors should go beyond a fragmented approach that focuses on single elements and pay more attention to aggregate behavioral profiles.

A key theoretical contribution is the differentiation between good dietary behavior and health-boosting physical activity. Although both are part of the broader domain of healthy lifestyles, empirical data reveal that they do not necessarily follow the same pattern at the macro level. This conclusion does not contradict research on healthy lifestyles but adds nuance, showing that different behaviors may follow distinct social, cultural, and institutional logics at the country level. The paper also confirms the usefulness of excess body weight as an intermediate predictor of health vulnerability while showing that this component alone cannot account for disparities between countries. The results on smoking also support the hypothesis of clustering of risk behavior, as smoking is more strongly associated with a poor dietary profile than with health outcomes in the short term. This research supports the view that population health should be understood as the result of behavioral and contextual systems rather than an aggregate of individual choices.

### 5.2. Practical Implications

The results of the study have substantial practical implications for the formulation of nutrition policy, obesity prevention initiatives, and health promotion activities in the European Union. First, the positive association between healthy eating behavior and life expectancy suggests that programs promoting daily fruit and vegetable consumption remain necessary. However, such regulations should not be limited to general information campaigns, as differences across countries suggest that pricing, the availability of healthy foods, nutrition education, and the overall food environment are critical factors. An effective approach should involve actions on prices, school meals, labeling, public procurement, the marketing of fresh products, and the reduction of barriers.

Second, the findings suggest that physical activity is associated with life expectancy but, on its own, does not explain differences in excess body weight. The conclusion suggests that food, urban, and social policies should include programming to promote movement. Better population health can be achieved through safe public areas, infrastructure for walking and cycling, community sport, physical exercise in schools, and the promotion of active transportation. However, their contribution to body weight will be limited unless the dietary environment encourages excessive consumption of energy-dense foods.

Thirdly, the study of clusters demonstrates that the lifestyle–health profile is not homogeneous across EU Member States. Uniform measures at the European level should therefore be accompanied by initiatives tailored to each country’s distinctive profile. Countries with poorer diets and higher smoking rates need coordinated efforts to reduce behavioral hazards. Preventive programs for long-term health are needed for countries with good longevity but substantial excess body weight. Countries with favorable dietary profiles should secure their existing advantages by promoting healthy, affordable diets. The key practical implication of this study is that the observed country-level patterns point to the potential value of differentiated, contextual, and intersectoral policy approaches, although the effectiveness of specific interventions cannot be established from the present cross-sectional analysis.

### 5.3. Limitations and Future Research

The paper should be read in light of key methodological limitations. The analysis is first cross-sectional and based on aggregate country-level data for 2019. Thus, the results reflect connections between national profiles rather than causal relationships between individual behaviors and health consequences. This also creates a risk of ecological fallacy, because associations observed at the country level cannot be assumed to apply to individuals within those countries. This limitation is particularly important for variables such as smoking and excess body weight, where the effects on longevity accumulate over lengthy periods and can manifest with long time lags. Second, the number of observations is limited, as the unit of analysis is the Member State of the European Union. With only 27 observational units, statistical power is reduced, confidence in weaker associations is limited, and the feasibility of testing more sophisticated models with several predictors is restricted. This is why the empirical results should be interpreted as exploratory country-level evidence rather than as definitive statistical confirmation.

Another limitation concerns the interpretation of nonsignificant results. Several associations, especially those involving sugar-sweetened soft drink consumption and healthy life years, were not statistically significant and should not be treated as meaningful trends. Future studies using individual-level data, repeated survey waves, or more detailed nutritional indicators could test whether the associations documented in clinical and epidemiological literature are also observable across countries after accounting for age, sex, socioeconomic status, total dietary sugar intake, and broader dietary patterns.

The absence of additional contextual variables also limits the cluster analysis. The identified typologies are based on lifestyle behaviors, excess weight, and population health outcomes, but they do not directly include socioeconomic inequalities, educational attainment, income levels, health system indicators, or measures of preventive care. As a result, the clusters should be interpreted primarily as descriptive profiles. Future research could combine lifestyle–health indicators with contextual variables such as education, income inequality, healthcare access, public health expenditure, social protection, and food-environment indicators to more clearly explain why countries follow different lifestyle–health trajectories.

Another restriction is due to the structure of the EHIS indicators, which include self-reported data for some of the behaviors studied and may therefore be affected by reporting bias. Fruit and vegetable intake, physical activity, smoking, and sweetened beverage intake may reflect cultural differences in reporting, recall, or social desirability. Self-perceived health is also a useful indicator, though it remains vulnerable to cultural norms and differing social expectations. Furthermore, the study employs rather general dietary markers and does not capture the full range of dietary quality, caloric intake, or consumption of ultra-processed items, fiber, salt, fats, or total sugar. More specifically, the healthy dietary behavior indicator is based only on daily fruit and vegetable consumption. Although these variables are harmonized and comparable across EU Member States, they cannot capture the complexity of dietary quality, meal composition, dietary traditions, meal timing, breakfast habits, beverage choices, or broader nutritional patterns.

The study also does not capture meal timing, breakfast consumption, full dietary patterns, sleep-related eating behaviors, or interactions between sleep and nutrition. These aspects are relevant for a more complete understanding of nutritional behavior, obesity risk, metabolic regulation, and population health, because chrononutrition research shows that food timing, circadian rhythm, time-restricted eating, and irregular or late meal patterns may influence energy balance, cardiometabolic regulation, weight gain, obesity risk, and sleep quality [68,69,70,71]. Future research could integrate such indicators if comparable cross-country data become available, to provide a more comprehensive assessment of nutrition-related determinants of health across EU Member States.

In addition, the available data do not include clinical biomarkers or direct measures of nutritional status. Future studies could therefore strengthen the disease-prevention perspective by incorporating updated, comparable indicators, such as fasting glucose, HbA1c, insulin resistance, lipid profile, inflammatory markers, blood pressure, vitamin D status, iron status, and other micronutrient-related biomarkers. Such variables would enable clearer distinction between dietary intake behaviors and nutrient status changes across different metabolic disease states, while also allowing a more clinically informative comparison among EU Member States.

Future research should make use of the new EHIS data as soon as they are available in the Eurostat Data Browser and in Eurostat Statistics Explained materials. The first partial results are expected in the second half of 2026. These will allow researchers to update the analysis and compare lifestyle–health characteristics between the 2019 wave and the new wave of data. One significant direction would be to conduct a time-series comparison to see whether the typologies found in this study endure, fade, or reconfigure. Adding economic and institutional variables, such as GDP per capita, health expenditure, education level, income inequality, or poverty, could also help explain why some countries achieve high health outcomes despite behavioral or metabolic vulnerabilities. Moreover, future studies could explore whether the associations observed at the national level also hold at the individual level, using individual-level microdata when they become available.

## 6. Conclusions

The study presents a comparative picture of the evolution of dietary habits, physical activity, smoking, sweetened beverage intake, excess body weight, and health outcomes throughout the Member States of the European Union. The results reveal that the link between lifestyle and health is not a unified European pattern, but rather several national profiles, each with its own combinations of advantages and risks. This discovery is important because it shows that community health cannot be described by a single nutritional, behavioral, or metabolic indicator in isolation.

A clearer link is shown between healthy dietary behavior and life expectancy, underlining the importance of fruit and vegetable consumption as an indicator of a positive nutritional profile. There is also a positive association between physical exercise and longevity. Nevertheless, the association with excess body weight is still less straightforward. Excess body weight is an essential indicator of population vulnerability, but its impacts must be read in the context of social, economic, and health-related situations. Smoking is identified by its relationship with poorer eating behavior, validating the clustering of lifestyle risks into wider behavioral profiles. Consumption of sweetened beverages did not play the expected explanatory role, suggesting that more extensive evaluations of the food environment and individual consumption are needed.

The main empirical contribution of the study is the identification of lifestyle–health typologies in EU countries. These typologies indicate that some countries are characterized by higher fruit and vegetable consumption and better longevity indicators, while others are characterized by higher levels of selected lifestyle risks and lower population health indicators. In contrast, some countries show mixed configurations of favorable and unfavorable indicators. These findings should be interpreted as country-level associations and descriptive patterns, not as evidence of the effectiveness of specific interventions. In this way, the article suggests that public health strategies may benefit from greater sensitivity to national contexts, behavioral configurations, and broader lifestyle–health patterns. However, further longitudinal, contextual, and individual-level research is needed before drawing firm conclusions about which interventions are most effective in improving population health outcomes.

## Figures and Tables

**Table 1:** Description of the variables used in the analysis.

Variable	Dataset	Measures	References
DFC	Daily fruit consumption	Percentage	[44]
DVC	Daily vegetable consumption	Percentage	[44]
HEPA	Health-enhancing physical activity	Percentage	[45]
DS	Daily smoking	Percentage	[46]
DDSD	Sugar-sweetened soft drink consumption	Percentage	[47]
BMIOV	Overweight prevalence	Percentage	[48]
BMIOB	Obesity prevalence	Percentage	[48]
LEB	Life expectancy at birth	Year	[49]
HLYB	Healthy life years at birth	Year	[49]

Source: authors’ design based on Eurostat [44,45,46,47,48,49].

**Table 2:** Descriptive statistics of the main variables.

Variable	Mean	SD	Min	Max
Daily fruit consumption	51.23	10.90	23.70	69.50
Daily vegetable consumption	48.46	12.63	17.40	78.40
Healthy dietary behavior	0.00	0.88	−2.49	1.55
Health-enhancing physical activity	49.71	23.98	9.90	95.40
Daily smoking	5.03	1.61	2.30	8.30
Sugar-sweetened soft drink consumption	8.37	4.03	2.40	20.40
Overweight prevalence	53.50	5.16	44.70	63.90
Obesity prevalence	17.47	3.95	10.50	28.10
Life expectancy at birth	80.49	2.78	75.10	84.00
Healthy life years	62.39	5.32	53.10	73.30

Source: authors’ design using SPSS v.27.0 (IBM Corporation, Armonk, NY, USA). Note: Healthy dietary behavior is a composite indicator computed as the arithmetic mean of standardized daily fruit consumption and standardized daily vegetable consumption. Therefore, its mean is approximately 0.00 by construction, while the standard deviation, minimum, and maximum values indicate cross-country variation around the EU average.

**Table 3:** Bivariate correlations between lifestyle behaviors, excess weight indicators, and population health outcomes.

Hypothesis	Variables	Pearson r	*p*	Spearman Rho	*p*
H1	HDB—LEB	0.459	0.016	0.361	0.064
H1	HDB—HLY	0.270	0.174	0.263	0.186
H1	HDB—SPH	0.104	0.605	0.189	0.346
H2	HEPA—LEB	0.435	0.023	0.323	0.101
H2	HEPA—BMIOV	−0.320	0.103	−0.313	0.112
H2	HEPA—BMIOB	0.001	0.996	0.051	0.801
H3	BMIOV—LEB	−0.505	0.007	−0.568	0.002
H3	BMIOV—HLY	−0.255	0.200	−0.374	0.054
H3	BMIOB—HLY	−0.221	0.268	−0.449	0.019
H4	DS—HDB	−0.572	0.002	−0.597	0.001
H4	DS—LEB	−0.174	0.386	−0.208	0.297
H5	DDSD—BMIOV	−0.002	0.994	0.137	0.495
H5	DDSD—LEB	0.113	0.576	0.000	0.999
H5	DDSD—HLY	0.289	0.144	0.364	0.062

Source: authors’ design using SPSS v.27.0 (IBM Corporation, Armonk, NY, USA).

**Table 4:** Reduced multiple regression models.

Dependent Variable	Predictors	R^2^	Adjusted R^2^	F	*p*
LEB	HDB, HEPA, BMIOV	0.474	0.405	6.900	0.002
HLY	HDB, HEPA, BMIOV	0.113	−0.003	0.977	0.421
LEB	HDB, HEPA, BMIOV, DS, DDSD	0.479	0.355	3.864	0.012
HLY	HDB, HEPA, BMIOV, DS, DDSD	0.175	−0.021	0.891	0.505

Source: authors’ design using SPSS v.27.0 (IBM Corporation, Armonk, NY, USA).

**Table 5:** Coefficients for the main reduced model predicting life expectancy.

Predictor	B	SE	Standardized Beta	t	*p*
Healthy dietary behavior	1.206	0.496	0.382	2.433	0.023
Health-enhancing physical activity	0.039	0.019	0.339	2.115	0.046
Overweight prevalence	−0.161	0.089	−0.298	−1.798	0.085

Source: authors’ design with SPSS v.27.0 (IBM Corporation, Armonk, NY, USA).

**Table 6:** Final standardized cluster centers.

Variable	Cluster 1	Cluster 2	Cluster 3
Daily fruit consumption	0.960	−0.804	0.484
Daily vegetable consumption	1.271	−0.508	−0.026
Health-enhancing physical activity	−0.377	0.162	−0.005
Daily smoking	−0.442	0.780	−0.716
Sugar-sweetened soft drink consumption	0.587	−0.486	0.289
Overweight prevalence	−1.271	0.061	0.562
Healthy life years	0.561	−0.648	0.497
Life expectancy	0.826	−0.490	0.174

Source: authors’ design using SPSS v.27.0 (IBM Corporation, Armonk, NY, USA).

**Table 7:** Differences between clusters based on K-means ANOVA output.

Variable	F	*p*
Daily fruit consumption	15.633	<0.001
Daily vegetable consumption	9.061	0.001
Health-enhancing physical activity	0.493	0.617
Daily smoking	12.766	<0.001
Sugar-sweetened soft drink consumption	3.142	0.061
Overweight prevalence	9.207	0.001
Healthy life years	6.457	0.006
Life expectancy	4.075	0.030

Source: authors’ design using SPSS v.27.0 (IBM Corporation, Armonk, NY, USA).

**Table 8:** Hypothesis assessment.

Hypothesis	Conclusion	Empirical Interpretation
H1	Partially supported	HDB is positively associated with LEB, but not significantly with HLY or SPH.
H2	Partially supported	HEPA is positively associated with LEB; its negative association with BMIOV is not statistically significant.
H3	Supported mainly for LEB	BMIOV is negatively and significantly associated with LEB. BMIOB is negatively associated with HLY in the Spearman analysis.
H4	Partially supported	Daily smoking is strongly and negatively associated with HDB, but not directly with health outcomes.
H5	Not supported as a direct association	DDSD does not show the expected adverse associations with excess weight or health outcomes.
H6	Supported	Three distinct lifestyle–health typologies of EU countries were identified.

## Data Availability

The original contributions presented in this study are included in the article. Further inquiries can be directed to the corresponding author. Research data are also publicly available: https://ec.europa.eu/eurostat/databrowser/view/hlth_ehis_fv1b/default/table?lang=en&category=hlth.hlth_det.hlth_cfv (accessed on 3 June 2026); https://ec.europa.eu/eurostat/databrowser/view/hlth_ehis_pe9e/default/table?lang=en&category=hlth.hlth_det.hlth_pha (accessed on 3 June 2026); https://ec.europa.eu/eurostat/databrowser/view/hlth_ehis_sk1e/default/table?lang=en&category=hlth.hlth_det.hlth_smok (accessed on 3 June 2026); https://ec.europa.eu/eurostat/databrowser/view/hlth_ehis_fv7e/default/table?lang=en&category=hlth.hlth_det.hlth_cfv (accessed on 3 June 2026); https://ec.europa.eu/eurostat/databrowser/view/hlth_ehis_bm1e__custom_21753872/default/table?page=time:2019 (accessed on 3 June 2026); https://ec.europa.eu/eurostat/databrowser/view/hlth_hlye__custom_21754142/default/table (accessed on 3 June 2026).

## Abbreviations

The following abbreviations are used in this manuscript:

HDB	healthy dietary behavior
HEPA	health-enhancing physical activity
DS	daily smoking
DDSD	daily drinking of sugar-sweetened soft drinks
BMIOV	overweight prevalence
BMIOB	obesity prevalence
LEB	life expectancy at birth
HLY	healthy life years
EHIS	European Health Interview Survey
